# A Sensitive Cloud Point Extraction–Spectrophotometric Determination of Vanadium Using Pyrogallol and Aliquat 336

**DOI:** 10.3390/ijms27146279

**Published:** 2026-07-14

**Authors:** Andrea Gajdošová, Petya Racheva, Antoaneta Saravanska, Jana Šandrejová, Kiril Gavazov

**Affiliations:** 1 Department of Analytical Chemistry, Institute of Chemistry, Faculty of Science, Pavol Jozef Šafárik University in Košice, Moyzesova 11, 04154 Kosice, Slovakia; andrea.gajdosova@student.upjs.sk (A.G.); jana.sandrejova@upjs.sk (J.Š.); 2 Department of Chemical Sciences, Faculty of Pharmacy, Medical University of Plovdiv, 120 Buxton Bros Str., 4004 Plovdiv, Bulgaria; petya.racheva@mu-plovdiv.bg (P.R.); antoaneta.saravanska@mu-plovdiv.bg (A.S.)

**Keywords:** vanadium, benzene-1,2,3-triol, cloud point extraction, mixed micelle system, ionic liquid, spectrophotometric determination, white analytical chemistry, RGBfast model

## Abstract

A novel centrifuge-less cloud-point extraction (CL-CPE) method based on pyrogallol (PG) was developed for the spectrophotometric determination of total vanadium. The method employs a mixed micelle-mediated extraction system comprising the nonionic surfactant Triton X-114 and the ionic liquid Aliquat 336 (A336), which serves as a source of monovalent cations capable of forming an ion pair with the anionic vanadium–PG chelate. The extracted species, (A336^+^)[V^IV^(OH)(PG)_2_], exhibits several absorption maxima (309, 362, and 436 nm), providing enhanced selectivity through appropriate wavelength selection according to the sample matrix. The principal absorption maximum occurs at 309 nm. At a sevenfold preconcentration factor, the method provides high sensitivity at this wavelength, with a molar absorptivity of 3.2 × 10^7^ L mol^−1^ cm^−1^, a limit of detection of 0.11 ng mL^−1^, and a Sandell sensitivity of 1.6 × 10^−3^ ng cm^−2^. The applicability of the method was demonstrated through the analysis of drinking water samples, spent vanadium catalyst materials, and vanadium-containing dietary supplements. The method was further evaluated using the RGBfast model, a white analytical chemistry assessment tool that integrates analytical performance, environmental sustainability, and practical and economic efficiency. The evaluation indicated a high overall whiteness score.

## 1. Introduction

Pyrogallol (PG) is a simple polyphenolic compound that has been used for centuries and continues to play an important role in modern science [1,2,3,4,5]. PG’s versatility has led to its utilization in a variety of fields, including biotechnology, nanoscience, medicine, and analytical chemistry.

PG is a widely employed reagent in spectrophotometric analysis owing to its ability to form metal complexes [6,7,8,9,10] and generate colored products via redox [11,12] and charge-transfer [13] reactions. In addition, PG serves as a precursor or structural scaffold for numerous established [14,15,16,17,18,19,20] and newly synthesized derivatives, including 4-[(4-fluorophenyl)diazenyl]-1,2,3-trihydroxybenzene [21], 4-(4-sulphophenylazo)pyrogallol [22], 4-(4-acetamidophenylazo)pyrogallol [23], and 4-(6-nitro-2-benzothiazolylazo)pyrogallol [24]. Both PG and its derivatives have been extensively utilized as analytical reagents for metal ions, biologically significant anions, food additives, and active pharmaceutical ingredients.

PG is regarded as one of the most sensitive spectrophotometric reagents for vanadium, particularly when used in conjunction with an auxiliary reagent (AR) that provides hydrophobicity and enhanced extraction efficiency. The complexation reaction between vanadium and PG in aqueous or ethanolic media proceeds effectively at approximately pH 6.0 [7,9,25,26]. Although the addition of an AR may or may not alter the optimum pH range for quantitative extraction, it invariably imparts distinctive characteristics to the extraction–chromogenic system. Specifically, by changing the microenvironment around the chromophore, AR can affect both the position of absorption maxima and sensitivity. For instance, the apparent molar absorptivity coefficient (*ε*) of V–PG–AR complexes can vary considerably, ranging from 4.2 × 10^3^ L mol^−1^ cm^−1^ when antipyrine is the AR [27,28], to 1.5 × 10^6^ L mol^−1^ cm^−1^ when *N*-hydroxy-*N*,*N*′-diphenylbenzamide is utilized [7]. The value of *ε* is also contingent on the specific extraction technique, the preconcentration factor, the fraction extracted, and, in certain cases, the oxidation state of vanadium.

It is generally accepted that V(III), V(IV), and V(V) can all form complexes with PG, with V(IV) acting as the central metal ion. Consequently, all three oxidation states can be determined without a preliminary oxidation or reduction step [25,26]. In essence, PG is believed to “buffer” vanadium in the V(IV) oxidation state during complex formation, thereby simplifying the analytical procedure. However, this assumption has not been fully validated, particularly in the presence of ARs, which likely play a stabilizing role on V(V) at certain pH values [7,28].

In this study, we investigated complex formation in an ionic liquid-based, mixed micelle-mediated, centrifuge-less cloud point extraction (IL-MM-CL-CPE) system comprising V(V), PG, Triton X-114 (TX-114), and Aliquat 336 (A336). The aim was to develop a competitive methodology for vanadium determination that is consistent with the three principal dimensions of white analytical chemistry—analytical performance (red), environmental sustainability (green), and practical/economic efficiency (blue) [29,30,31,32].

**Figure 1:**
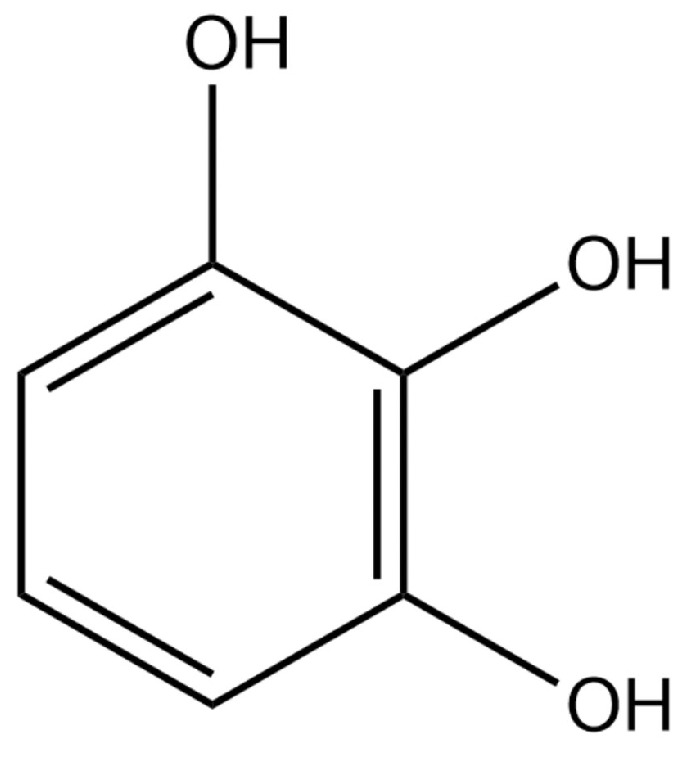
Chemical formula of pyrogallol (benzene-1,2,3-triol).

Mixed micelle-mediated cloud-point extraction (MM-CPE) and ionic-liquid-based cloud point extraction (IL-CPE) have emerged as promising approaches for enhancing analytical performance [33,34,35,36,37]. Centrifuge-less cloud-point extraction (CL-CPE) offers the additional advantage of eliminating the centrifugation step; however, this simplification is typically accompanied by longer phase-separation times [36,37,38,39,40,41,42], which may limit sample throughput. In our previous MM-CPE study employing 4-nitrocatechol, efficient extraction of vanadium was achieved without centrifugation after a 70 min incubation period [38]. In the present work, this limitation was largely overcome through the use of PG, which reduced the required incubation time by approximately 50%. This improvement may be attributed to the presence of a third hydroxyl group in PG (Figure 1), which does not participate directly in metal coordination but may form hydrogen bonds with the polyoxyethylene chains of TX-114. Such interactions could increase the affinity of the complex for the micellar phase, thereby enhancing extraction efficiency and facilitating phase separation [43,44].

## 2. Results and Discussion

### 2.1. Optimization Experiments

#### 2.1.1. Absorption Spectra

**Figure 2:**
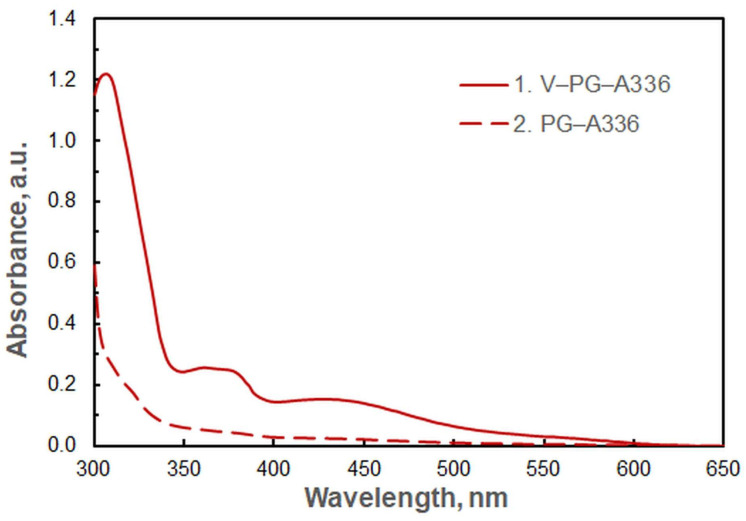
UV-Vis absorption spectra of the V−PG−A336 complex (1) and the blank (2). *c*_V_ = 3.6 × 10^−8^ mol L^−1^, *c*_PG_ = 1.43 × 10^−2^ mol L^−1^, *c*_A336_ = 7.1 × 10^−4^ mol L^−1^, *w*_TX-114_ = 2.1%, pH = 3.4, *V*_buffer_ = 1 mL, incubation time (*t*_inc_) of 30 min at 70 °C.

Figure 2 shows the absorption spectra of the complex against a blank (1) and of the blank against water (2). The spectrum of the complex displays multiple absorption maxima, with their positions remaining practically constant under variations in pH. The principal absorption maximum is located at 309 nm. Additional maxima are observed at approximately 362, 374, and 436 nm.

It is noteworthy that the principal maximum at 309 nm has not been previously documented for any V–PG-containing complex. The same applies to the absorption maxima observed at 362 and 374 nm. The extant literature contains reports of maxima of V–PG–AR complexes at 533 nm [9], 430–440 nm [7], 595–600 nm [45,46], and 630–640 nm [27,28].

#### 2.1.2. Effects of pH and Buffer’s Volume

**Figure 3:**
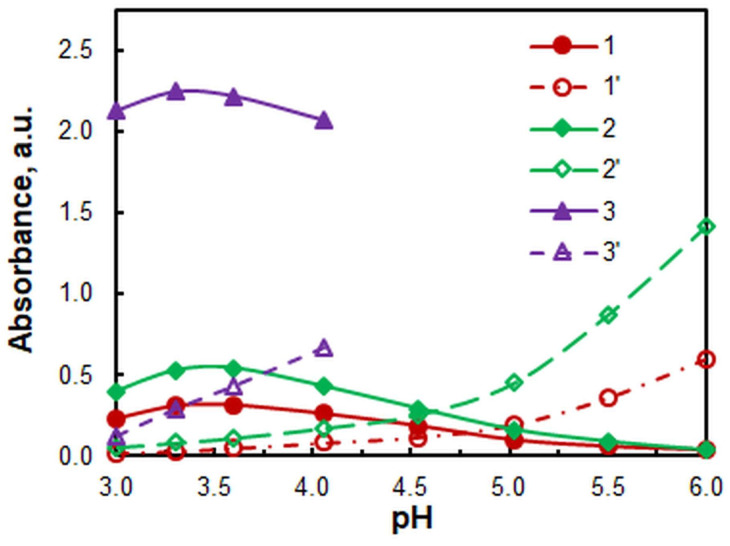
The effect of pH at 436 nm (1, 1′), 362 nm (2, 2′), and 309 nm (3, 3′). *c*_V_ = 7.1 × 10^−8^ mol L^−1^ (1–3), *c*_PG_ = 1.43 × 10^−2^ mol L^−1^, *c*_A336_ = 7.1 × 10^−4^ mol L^−1^, *w*_TX-114_ = 2.1%, *V*_buffer_ = 1 mL; *t*_inc_ = 30 min at 70 °C.

The effect of pH on the absorbance of the complex (1–3) and corresponding blank (1′–3′) at 436 nm (1, 1′), 362 nm (2, 2′), and 309 nm (3, 3′) is shown in Figure 3. The pH was maintained using buffer solutions prepared by mixing 2.0 mol L^−1^ ammonium hydroxide and acetic acid. The absorbance profiles recorded at 309, 362, and 436 nm exhibited similar behavior, with maximum absorbance observed in the pH range of 3.3–3.6. Accordingly, all subsequent experiments were performed at pH 3.3–3.4, where the highest selectivity and lowest blank absorbance were achieved.

**Figure 4:**
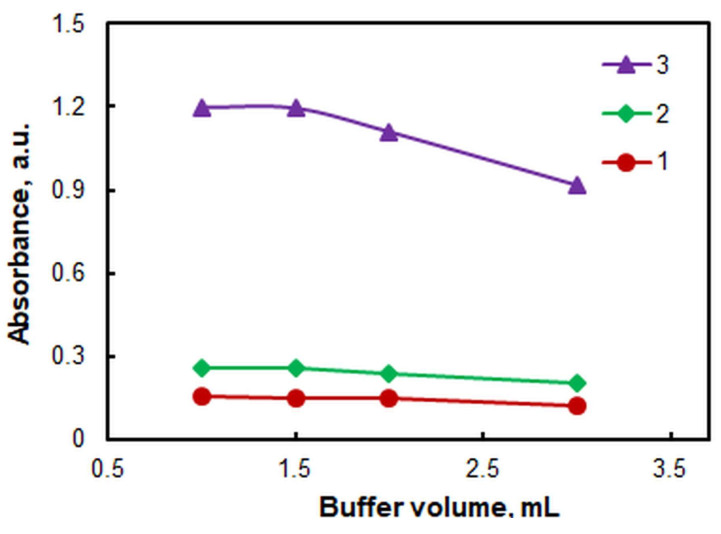
The effect of buffer volume (pH 3.3) at 436 nm (1), 362 nm (2), and 309 nm (3). *c*_V_ = 3.6 × 10^−8^ mol L^−1^, *c*_PG_ = 1.43 × 10^−2^ mol L^−1^, *c*_A336_ = 7.1 × 10^−4^ mol L^−1^, *w*_TX-114_ = 2.1%, pH 3.3, *t*_inc_ = 30 min at 70 °C.

The effect of buffer volume was investigated using a pH 3.3 buffer (Figure 4). It is noteworthy that effective gravitational (centrifuge-less) phase separation was not achieved when the buffer volume was ≤0.5 mL. Subsequent studies were conducted in the presence of 1.0 or 1.5 mL of the buffer. At these volumes, the analytical signal reached a maximum.

#### 2.1.3. Effect of PG and A336 Concentrations

**Figure 5:**
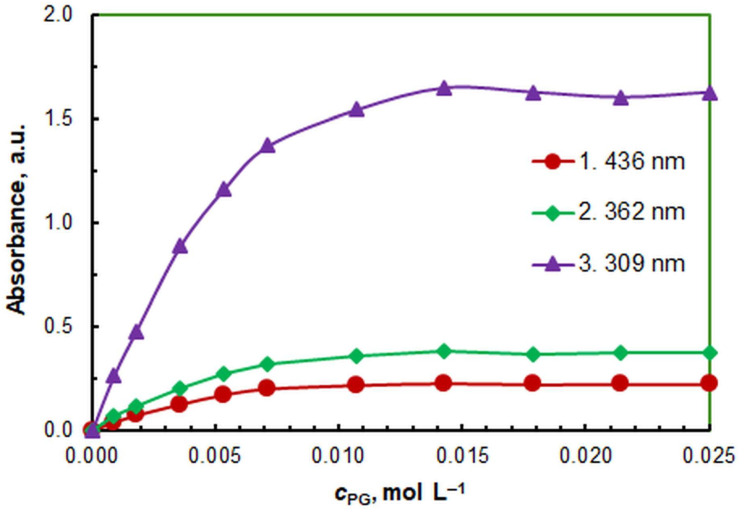
The effect of PG concentration. *c*_V_ = 5 × 10^−8^ mol L^−1^, *c*_A336_ = 7.1 × 10^−4^ mol L^−1^, *w*_TX-114_ = 2.1%, pH = 3.3, *t*_inc_ = 30 min at 70 °C.

**Figure 6:**
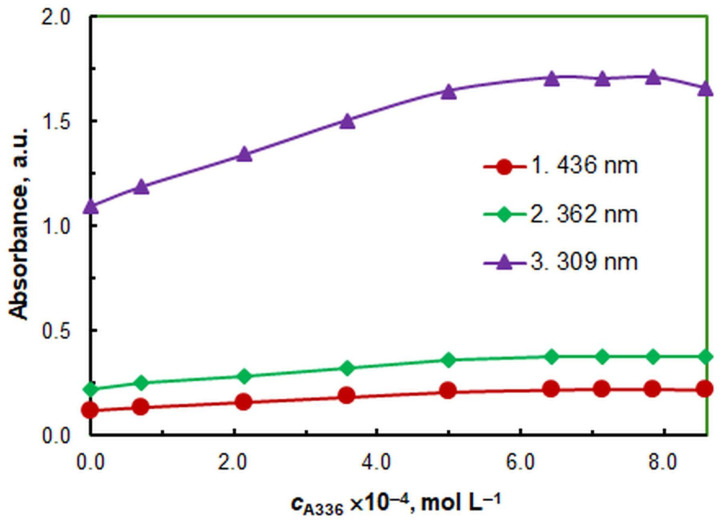
The effect of A336 concentration. *c*_V_ = 5 × 10^−8^ mol L^−1^, *c*_PG_ = 1.43 × 10^−2^ mol L^−1^, *w*_TX-114_ = 2.1%, pH = 3.3, *t*_inc_ = 30 min at 70 °C.

The effects of PG and A336 concentrations on the absorbance are shown in Figure 5 and Figure 6, respectively. It is noteworthy that the analytical signal at *c*_A336_ = 0 is not zero (Figure 6). This behavior is not unusual for extraction systems involving ternary complexes [28,38,45], including those employing A336 as a cationic ion-association reagent [39,40]. Subsequent studies were carried out at *c*_PG_ = 1.43 × 10^−2^ mol L^−1^ and *c*_A336_ = 7.1 × 10^−4^ mol L^−1^.

#### 2.1.4. Effect of TX-114 Mass Fraction

**Figure 7:**
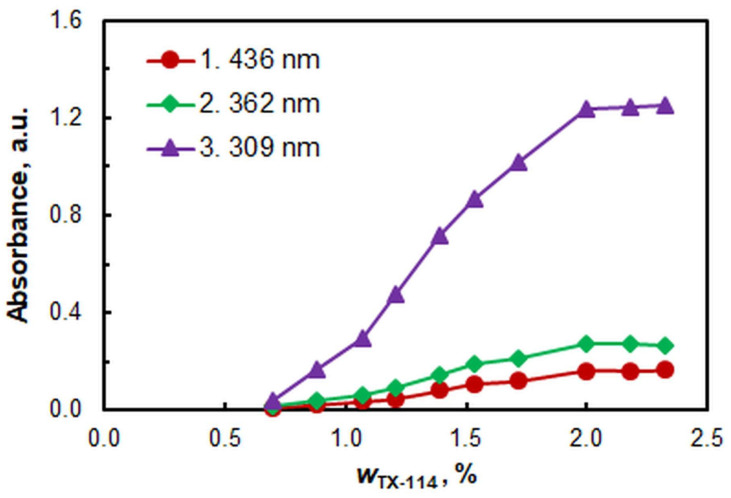
The effect of TX-114 mass fraction. *c*_V_ = 3.6 × 10^−7^ mol L^−1^, *c*_PG_ = 1.43 × 10^−2^ mol L^−1^, *c*_A336_ = 7.1 × 10^−4^ mol L^−1^, pH = 3.3, *t*_inc_ = 30 min at 70 °C.

The effect of the TX-114 mass fraction (*w*_TX-114_) is shown in Figure 7. Maximum extraction was achieved at *w*_TX-114_ ≥ 2.0%. Therefore, a mass fraction of 2.1% was selected for all subsequent experiments to ensure robust operating conditions.

#### 2.1.5. Mixed Surfactant Solution

After establishing the optimum levels of A336 and TX-114, the two surfactants were combined into a single stock solution. The extraction results obtained with the mixed surfactant solution were essentially identical to those achieved when the surfactants were added separately. However, the use of a mixed solution offers several practical advantages:

1. Simplified experimental procedure—Fewer solutions need to be prepared, stored, and handled, resulting in a more streamlined workflow.
2. Elimination of organic solvents—In the presence of TX-114, A336 becomes readily soluble in water, eliminating the need for an organic solvent.
3. Reduced reagent volume—The volume of the combined surfactant solution can be optimized, thereby increasing the proportion of sample solution that can be accommodated in the extraction system.

The mixed aqueous solution of A336 and TX-114 used in subsequent experiments was prepared to contain 5.0 × 10^−3^ mol L^−1^ A336 and 15% TX-114.

#### 2.1.6. Effect of Incubation Temperature

The effect of incubation temperature was evaluated at 60 °C, 70 °C, and 80 °C. The absorbance measured at 70 °C was approximately 14% higher than that obtained at 60 °C and 5% higher than that obtained at 80 °C. Accordingly, an incubation temperature of 70 °C was selected for all subsequent experiments.

#### 2.1.7. Effect of Incubation Time

**Figure 8:**
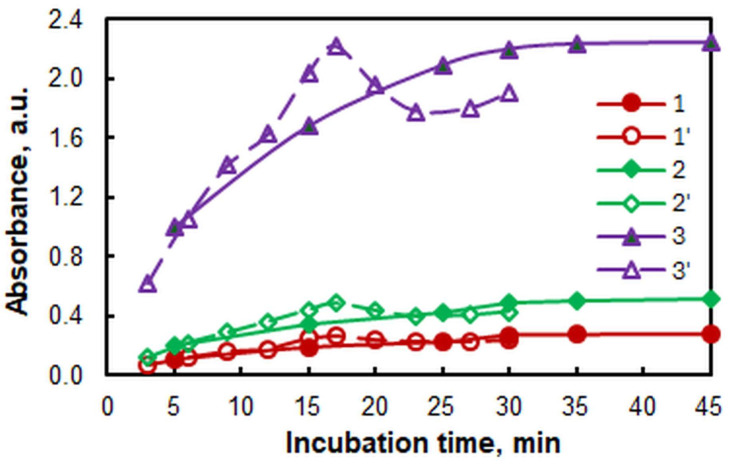
The effect of incubation time at 70 °C. *c*_V_ = 7.1 × 10^−8^ mol L^−1^, *c*_PG_ = 1.43 × 10^−2^ mol L^−1^, *c*_A336_ = 7.1 × 10^−4^ mol L^−1^, *w*_TX-114_ = 2.1%, pH = 3.3. *λ* = 436 nm (1, 1′), 362 nm (2, 2′), and 309 nm (3, 3′).

The effect of incubation time at 70 °C is shown in Figure 8. Two heating methods were compared: a conventional water bath (1–3) and an ultrasonic bath (1′–3′). In the water-bath system, maximum absorbance was attained after an incubation time (*t*_inc_) of 30 min or longer. In contrast, the ultrasonic bath enabled a substantially shorter incubation period (*t*_inc_, _US_). However, this approach exhibited certain limitations, including the presence of a pronounced maximum at approximately 17 min and poorer reproducibility.

#### 2.1.8. Cooling Time, Phase Separation, and Surfactant-Rich Phase (SRP) Treatment

The samples were cooled in a freezer maintained at −20 °C. Under these conditions, 25 min was sufficient to increase the viscosity of the SRP, thereby enabling the easy removal of the upper aqueous phase by decantation. Following phase separation, the SRP was treated with 300 μL of ethanol and diluted with deionized water to a final volume of 2.0 mL.

Ethanol was selected as the viscosity-reducing agent in preference to methanol because of its lower weighted hazards number (WHN) [31,47] and its successful application in previous studies involving metal complexes extracted with A336 or PG [9,10,39,40,48]. The presence of ethanol was found not to affect the absorbance values. In principle, the SRP could be supplemented with deionized water alone (without ethanol). However, this would result in a slower procedure, as additional heating and more vigorous shaking would be required for homogenization [49].

Table 1 summarizes the optimized experimental parameters. Under the selected conditions, the absorbance remained stable for at least 1 h. This stability was confirmed by recording the spectra of both the sample and the corresponding blank (see Figure 2) at 5 min intervals over a 1 h period.

### 2.2. Formula and Chemical Equations

#### 2.2.1. Stoichiometric Ratios and Vanadium Oxidation State

**Figure 9:**
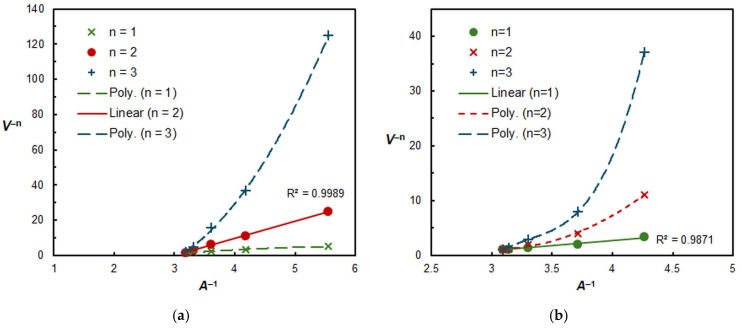
Determination of the PG:V (**a**) and A336:V (**b**) molar ratios by the straight-line Asmus method. *λ* = 436 nm.

The straight-line method [50] (Figure 9) and the method of mobile equilibrium [51] were used to determine the PG-to-vanadium and A336-to-vanadium molar ratios in the ternary complex. Both methods yielded identical results at all three investigated wavelengths, namely PG:V = 2:1 and A336:V = 1:1. The combined results indicate that the ternary complex has a stoichiometric composition of 1:2:1 (V:PG).

This stoichiometry may be interpreted in two ways:

1. Vanadium retains its +5 oxidation state during complex formation. In this case, the extracted species can be represented as (A336^+^)[V^V^O(PG)_2_]. A similar complex has been reported for the V(V)–4-nitrocatechol–cetylpyridinium chloride system [38]. However, in that system the catechol derivative contains an electron-withdrawing nitro group, which reduces its tendency to participate in redox processes.
2. V(V) is reduced to V(IV) by PG during the complex formation process. We consider this interpretation to be the most plausible because it is consistent with both the experimental observations and the theoretical background:
  (A) The absorption spectra obtained for V(V) and V(IV) are virtually identical.
  (B) Complex formation occurs under more acidic conditions than those reported for other extraction systems [9,25,26], an environment known to favor the reduction of V(V) species [28,52,53].
  (C) Under optimal conditions, PG is present in substantial excess, providing sufficient reducing capacity to facilitate the reduction of V(V) to V(IV).

Based on these considerations, we suggest that the extracted species is most likely formed through the reduction of V(V) to V(IV) during the complexation process.

#### 2.2.2. Equations of Complexation and Extraction

The reagent A336 acts as a source of monovalent cations (A336^+^). Consequently, to satisfy the experimentally established molar ratio of 1:1 (V:A336), the extracted ion pair must contain a monovalent complex anion. The formation of this anion is represented by Equation (1), in which pyrogallol (PG) is denoted as C_6_H_3_(OH)_3_. The proposed reaction is consistent with literature reports identifying VO_2_^+^ as the predominant V(V) species in acidic aqueous solutions [53,54].

The extraction of the ternary complex into the SRP is described by Equation (2). It is most likely that the two adjacent hydroxyl groups in each pyrogallol ligand undergo deprotonation and participate in coordination to the vanadium center, forming chelate rings. The third hydroxyl group of each pyrogallol moiety is presumed to remain protonated and non-coordinated. This group may enhance the affinity of the complex for the micellar phase through hydrogen bonding with Triton X-114, whose oxygen-containing polyoxyethylene chains are strong hydrogen-bond acceptors. The hydroxyl group coordinated to the vanadium center may likewise contribute to these interactions.

V^V^O_2_^+^ + 2C_6_H_3_(OH)_3_ + e^−^ = [V^IV^(OH)(OO(OH)C_6_H_3_)_2_]^−^ + H_2_O + H^+^ (1)

V^V^O_2_^+^_(aq)_ + 2C_6_H_3_(OH)_3(aq)_ + e^−^_(aq)_ + A336^+^_(aq)_ = (A336^+^)[V^IV^(OH)(OO(OH)C_6_H_3_)_2_]_(SRP)_ + H_2_O + H^+^_(aq)_ (2)

### 2.3. Analytical Characteristics

Calibration graphs were constructed under optimal conditions using vanadium standard solutions in the concentration range 0.55–7.2 ng mL^−1^ (*n* = 10). Linear responses were obtained up to 3.6 ng mL^−1^ V (*n* = 6). The regression parameters at the three analytical wavelengths (309, 362, and 436 nm), together with other relevant analytical figures of merit, are summarized in Table 2.

Table 2 also allows comparison between conventional water-bath heating and ultrasonic-assisted (UA) extraction. The slopes of the calibration curves are essentially identical for both methods, indicating comparable analytical sensitivity. However, ultrasonic heating leads to slightly decreased repeatability and results in higher limits of detection (LOD) and quantification (LOQ). In all cases, the intercepts of the regression lines are slightly negative but not statistically different from zero.

The preconcentration factor was calculated as the ratio of the sample volume (14 mL) to the final volume of the diluted SRP (*V*_SRP_ = 2.0 mL), yielding a value of 7.0.

The robustness was evaluated by introducing small, deliberate variations in several experimental parameters. The factors investigated included: (i) pH (3.3 and 3.4), (ii) PG concentration (1.43 × 10^−2^ and 1.79 × 10^−2^ mol L^−1^), (iii) the residence time of the PG solution (freshly prepared or stored for one day), (iv) the mass fraction of TX-114 (2.00% and 2.14%), and (v) the use of separate solutions of TX-114 (aqueous) and A336 (methanolic) versus a combined aqueous solution. The results demonstrated that these small variations had no significant effect on the analytical performance, confirming that the proposed procedure is robust and reliable under normal laboratory operating conditions.

### 2.4. Effect of Foreign Ions

The effect of various ions on the determination of vanadium is summarized in Table 3. The presence of absorption maxima at different wavelengths provides an opportunity to select the most appropriate analytical wavelength according to the sample matrix. For example, 309 nm is the preferred wavelength for samples containing significant amounts of Mo(VI), whereas 436 nm affords more accurate results in the presence of W(VI). Notably, both ions have been reported as major interferents in another PG-based method for vanadium determination [25].

In general, the proposed method exhibits high selectivity at all three wavelengths. In addition to common inorganic ions, including alkali and alkaline earth metal ions, chloride, sulfate, and nitrate ions, it is highly tolerant of several d-block ions, such as Cd(II), Co(II), Hg(II), Mn(II), Ni(II), and Zn(II), as well as the post-transition metal Pb(II) and the f-element U(VI).

The method is also tolerant of moderate amounts of Al(III), I^−^, Cr(III), and Re(VII). A 100-fold excess of Ge(IV) affects the determination only at 362 nm, while no interference is observed at the other wavelengths.

The most significant interferences arise from Cu(II) and Fe(III). As demonstrated, a tenfold excess of Fe(III) can be effectively masked with H_2_PO_2_^−^ or F^−^. Despite extensive efforts, attempts to mask Cu(II) using tartrate, citrate, or thiourea were unsuccessful. This limitation may be addressed in future studies through a more detailed investigation of the Cu–PG–A336 complex and by applying the approaches described in [55,56].

### 2.5. Analytical Application

The applicability of the developed method was assessed by analyzing vanadium-containing dietary supplements, drinking water samples, and spent vanadium catalysts from sulfuric acid production. The results obtained for the dietary supplements at the three absorption maxima (Table 4) were statistically indistinguishable and in good agreement with the vanadium content declared by the manufacturer.

The analytical results for the drinking water and catalyst samples are presented in Table 5 and Table 6, respectively. The results for the catalyst samples were in good agreement with those obtained using an alternative method [56] and exhibited better repeatability.

### 2.6. Comparison with Other Methods for Vanadium Determination

Table 7 compares the key analytical characteristics of the proposed method with those of previously reported spectrophotometric methods. The present method is characterized by high sensitivity, simplicity, and cost-effectiveness. The LOD and LOQ values are very low, particularly at 309 nm (0.11 and 0.36 ng mL^−1^, respectively). The complex exhibits several absorption maxima across the UV-Vis spectral region, enabling improved selectivity toward various concomitant ions. The method is robust, as most operating parameters can vary over relatively wide ranges without significantly affecting the analytical signal. In addition, the results obtained for V(V) and V(IV) are virtually identical, enabling the determination of total vanadium without additional oxidation or reduction steps.

Unlike conventional CPE methods, the proposed procedure does not require centrifugation. The addition of salting-out agents to enhance extraction efficiency, as employed in methods [9,57,58], is likewise unnecessary. The reagents employed are inexpensive and readily available, and no reagent synthesis is required, unlike in several previously reported methods [24,59,60,61,62,63]. Moreover, the proposed method requires only a small amount of organic solvent (300 μL ethanol per sample). In this respect, it offers an advantage not only over liquid–liquid extraction methods [7,61,64,65,66], but also over methods based on dispersive liquid–liquid semi-microextraction [62], microextraction with solidification of a floating organic drop [66], and solid-phase extraction [67].

Compared with other reported CL-CPE methods [38,68], the proposed procedure offers the advantage of a relatively short incubation time. Furthermore, when rapid analysis is required, the incubation time can be further reduced by applying ultrasound-assisted heating.

**Table 7:** Comparison with other spectrophotometric methods for the determination of vanadium.

Reagent(s)	Extraction Procedure	Extractant	Linear Range, μg L^−1^	LOD, μg L^−1^	*λ*_max_, nm	*ε*·10^−5^, L mol^−1^ cm^−1^	pH	Sample	Ref.
4NC + CPC	MM-CL-CPE	TX-114	2–305	0.6	670	1.22	5.5	Mineral water, spent catalyst, and dietary supplement	[38]
APANOL	–	–	100–4000	NR	533	0.102	3.0	Rice and flour	[63]
Br-PADAP	CPE	TX-114	7–300	2.2	603	NR	3.5	Tap water and river water	[55]
CHMFC	LLE	Dichloromethane	Up to 1100	55.9	425	0.6	0.2 mol L^−1^ CH_3_COOH	Synthetic and technical samples	[61]
DHMPhB	DLLSME	chloroform + methanol	13–612 V(IV,V) 38–1200 V(V)	3.9 V(IV,V) 11.4 V(V)	520 525	0.95 (V^IV^) 1.0 (V^V^)	4.0	Model mixtures and tap water	[62]
EDTA + Safranin T	UA-CPE	TX-114	2–180 (V^V^) 1–40 (V^IV^)	0.53 (V^V^) 0.26 (V^IV^)	530	NR	4.0 (V^V^) 5.0 (V^IV^)	Vegetal oils and vinegar	[58]
GB + KBrO_3_	–	–	1–100	0.31	537	NR	2.0	Water samples	[69]
HTAR + H_2_O_2_	CL-CPE	TX-100	Up to 510	0.8	582	1.66	7 × 10^−3^ mol L^−1^ H_2_SO_4_	Lake water and spent catalyst samples	[49]
HTAR + TTC	LLE	Chloroform	15–2000	4.6	549	0.52	4.7	Spent catalysts and pharmaceuticals	[64]
ICQ	LLE	Chloroform	Up to 7000	210	407	0.0738	2 mol L^−1^ CH_3_COOH	Synthetic mixtures	[66]
*N*-BPHA	LLE	Chloroform	Up to 1500	NR	530	0.0545	≥2.4 mol L^−1^ HCl	Natural waters	[65]
NTA8HQ + BIABP + H_2_O_2_	CPE	TX-114	1–70 (V^V^) 10–100 (V^IV^)	0.72 (V^V^) 1.78 (V^IV^)	634 625	16.0 (V^V^) 20.6 (V^IV^)	3.0	Water, soil, rice, and vegetables	[60]
PAR	MCPE	TX-114	50–600	5.51	568	0.185	5.5	Tap water	[57]
PG	–	–	Up to 14,000	47	580	0.0775	6.0	Synthetic samples	[25]
PG + HDPBA	LLE	Chloroform	Up to 40	1.5	430	15	4.5	Sea water, wastewater, cement dust, coal ash	[7]
PG + Safranin T	UA-CPE	TX-114	2–500	0.58	533	NR	6.0	Beverage samples	[9]
TA + CTAB	IP-SA-DLLME-SFOD	1-Undecanol	6–1000	1.8	600	NR	6.0	Fruit juice samples	[70]
TAN + H_2_O_2_	MWA-CL-CPE	TX-100	Up to 760	1.4	607	0.884	5 × 10^−3^ mol L^−1^ H_2_SO_4_	Natural water, aluminum alloy, catalyst, and solution for infusion	[56]
PG + A336	IL-MM-CL-CPE	TX-114	Up to 3.6	0.11 0.12 0.15	309 362 436	320 7.2 4.1	3.4	Water samples, dietary supplements, and spent catalyst	This work

Abbreviations: 4NC, 4-nitrocatechol; A336, Aliquat 336; APANOL, 1-[(4-antipylazo)]-2-naphthol; BDTA, benzyldimethyltetradecyleammonium chloride; BIABP, 2-[(benzoimidazolyl)azo]-4-benzyl phenol; Br-PADAP, 2-(5-bromo-2-pyridylazo)-5-diethylaminophenol; CHMFC, 6-chloro-3-hydroxy-7-methyl-2-(2′-furyl)-4H chromen-4-one; CL-CPE, centrifuge-less CPE; CPC, cetylpyridinium chloride; CTAB, cetyltrimethylammonium bromide; DHMPhB, 6,7-dihydroxybenzopyrylium bromide; DLLSME, dispersive liquid–liquid semi-microextraction; GB, Gallamine blue; HDPBA, *N*-hydroxy-*N*,*N*′-diphenylbenzamide; HTAR, 6-hexyl-4-(2-thiazolylazo)-resorcinol; ICQ, iodochlorhydroxyquin; IP-SA-DLLME-SFOD, ion pair-based surfactant-assisted dispersive liquid–liquid microextraction with solidification of floating organic drop; LLE, liquid–liquid extraction; MCPE, micro-CPE; MWA-CL-CPE, microwave-assisted centrifuge-less CPE; MM-CL-CPE, mixed-micelle-mediated centrifuge-less CPE; *N*-BPHA, *N*-benzoyl-*N*-phenylhydroxylamine; NR, not reported; NTA8HQ, 2-[2-(5-nitrothiazolyl)azo]-8-hydroxyquinoline; PG, pyrogallol; TA, tannic acid; TAN, 1-(2-thiazolylazo)-2-naphthol; TX-100, Triton X-100; TX-114, Triton X-114; UA-CPE, ultrasonic-assisted CPE.

### 2.7. Evaluation of the Method Using the RGBfast Model

The RGBfast model [31] was applied to evaluate the proposed PG–A336 method. This model is based on the concept of white analytical chemistry (WAC), in which greenness represents one of the three primary components of analytical whiteness. The remaining components are red, which reflects the analytical performance of the method in terms of trueness, precision, and sensitivity, and blue, which is associated with its practicality and economic efficiency.

Several spectrophotometric methods for vanadium determination, based on different extraction techniques, were selected for comparison. These included liquid–liquid extraction (LLE) [66], dispersive liquid–liquid semi-microextraction (DLLSME) [62], ion pair-based surfactant-assisted dispersive liquid–liquid microextraction with solidification of floating organic drop (IP-SA-DLLME-SFOD) [70], and microwave-assisted centrifuge-less cloud point extraction (MWA-CL-CPE) [56]. Since some of the parameters required for RGBfast evaluation were not specified in the original publications, approximate values were used where necessary. This limitation arises primarily from the difficulty of directly comparing different analytical targets, as well as from uncertainties associated with estimating the maximum analytical throughput (i.e., the maximum number of samples that can be processed per day).

**Figure 10:**
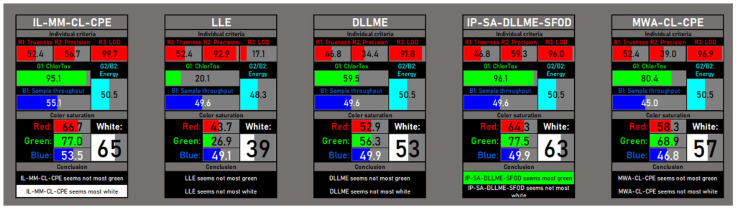
Evaluation of the analytical methods using the RGBfast model.

As shown in Figure 10, the IL–MM–CL-CPE method proposed in this work performs only slightly worse than the IP-SA-DLLME-SFOD method in terms of greenness. However, it compensates for this difference through its performance in the other evaluation criteria, resulting in the highest overall whiteness score among the methods compared.

## 3. Materials and Methods

### 3.1. Reagents and Solutions

All reagents were used without further purification. Their aqueous solutions were prepared using deionized water (18.2 MΩ·cm, ELGA-Veolia LabWater, High Wycombe, UK).

A V(V) stock solution (2.0 × 10^−4^ mol L^−1^) was prepared from ammonium metavanadate (puriss. p.a., Merck, Schnelldorf, Germany). Working solutions were obtained by appropriate dilution of the stock solution.

An aqueous solution of PG (puriss. p.a., Loba Feinchemie GmbH, Fischamend, Austria) was prepared at a concentration of 2.5 × 10^−1^ mol L^−1^ in the presence of hydrochloric acid (3 mL of 0.1 mol L^−1^ HCl per 50 mL of PG solution). The PG solution was stored in a dark container and used either on the day of preparation or on the following day.

TX-114 (laboratory grade, Merck, Schnelldorf, Germany) was used as aqueous solutions with mass fractions of 10% or 15%. The ionic liquid A336 (Merck, Schnelldorf, Germany) was dissolved either in methanol to obtain a 1.0 × 10^−2^ mol L^−1^ solution or in water containing TX-114. The mixed aqueous A336–TX-114 solution was prepared at a concentration of 5.0 × 10^−3^ mol L^−1^ with respect to A336 and a mass fraction of 15% with respect to TX-114.

Buffer solutions in the pH range 3.0–6.0 were prepared by mixing 2.0 mol L^−1^ solutions of ammonia and acetic acid.

### 3.2. Instrumentation

UV–Vis absorbance measurements were performed using Ultrospec 3300 Pro and Spectronic Camspec M550 spectrophotometers (Garforth, UK), both equipped with 10 mm semimicro cuvettes of 1.4 mL capacity. Cloud-point extraction experiments were carried out using a GFL 1023 water bath (GFL Gesellschaft für Labortechnik GmbH, Berlin, Germany). Ultrasound-assisted experiments were conducted using an Elmasonic Easy 120 H ultrasonic bath (Elma Schmidbauer GmbH, Singen, Germany) operating at a frequency of 37 kHz and an effective power of 200 W. pH measurements were performed with a WTW InoLab 7110 pH meter (Wissenschaftlich-Technische Werkstätten, Weilheim, Germany).

### 3.3. Sample Preparation

The dietary supplements Chromium & Vanadium (Natural Factors^®^, Coquitlam, Canada) and Vanadium, Vanadyl Sulfate with Chromium (Nutricost^®^, Vineyard, UT, USA) were purchased from a local pharmacy. According to the manufacturers’ specifications, each Chromium & Vanadium capsule contains 25 μg of vanadium in the form of citrate, whereas each Vanadium, Vanadyl Sulfate with Chromium capsule contains 2 mg of vanadium as VOSO_4_. Sample preparation was carried out according to previously reported procedures [38,68], without the oxidation step commonly required for the conversion of V(IV) to V(V). The resulting solution of the second dietary supplement was further diluted with water to adjust the vanadium concentration within the linear range of the calibration curve.

Spent V_2_O_5_–SiO_2_–K/Na catalyst materials used in sulfuric acid production were obtained from Holding KCM 2000, Plovdiv, Bulgaria. Sample preparation was carried out according to the procedure described in [68]. The resulting solutions were further diluted with water to bring the vanadium concentration within the linear range of the calibration curve.

Bottled table water intended for daily consumption (Gorna Banya, Serdika^®^, Sofia, Bulgaria) was purchased from a local supermarket. Spring water was collected from a mountain spring in the municipality of Batak, Bulgaria. A low-mineral-content water sample, not intended for daily consumption, was also collected directly from a spring (Bratsigovo, Bulgaria). Aliquots of 5.0 mL were used for water analyses.

### 3.4. Optimization Procedure

Solutions of TX-114, V(V), buffer, PG, and A336 were transferred into 15 mL centrifuge tubes. The mixtures were diluted with water to a final volume of 14 mL and incubated for 5–45 min in either a water bath or an ultrasonic bath at 60–80 °C. The tubes were then briefly cooled under running water and subsequently stored in a freezer at −20 °C for 25–30 min to facilitate phase separation and removal of the upper aqueous phase by decantation.

After phase separation, ethanol (300 μL) and deionized water were added to the viscous SRP. The mixtures were shaken to achieve homogenization, and the final volume was adjusted to 2.0 mL. The resulting solutions were then transferred to cuvettes for absorbance measurements.

### 3.5. Recommended Analytical Procedure

A 2.0 mL portion of the mixed surfactant solution (15% TX-114 and 5.0 × 10^−3^ mol L^−1^ A336) was transferred into a 15 mL conical-bottom centrifuge tube. An aliquot of the sample solution containing vanadium (not exceeding 3.6 ng mL^−1^), 1.0 mL of pH 3.4 buffer solution, and 0.8 mL of 2.5 × 10^−1^ mol L^−1^ PG solution were subsequently added. The mixture was diluted with water to a final volume of 14 mL and thoroughly mixed.

The tubes were incubated in a water bath at 70 °C for 30–35 min. Alternatively, an ultrasonic bath could be used under the same temperature conditions to reduce the required incubation time to 17 min. After incubation, the tubes were cooled under running water and then placed in a freezer at −20 °C for 25–30 min to facilitate phase separation. The upper aqueous phase was removed by decantation, leaving the SRP.

To the SRP, 300 μL of ethanol was added, followed by an appropriate amount of deionized water to adjust the final volume of the homogenized solution to 2.0 mL. The solution was mixed thoroughly and transferred into a 10 mm semimicro cuvette.

The absorbance was measured at 309, 362, or 436 nm against a reagent blank prepared in the same manner, except without addition of vanadium. The vanadium concentration was determined from the corresponding calibration curve.

## 4. Conclusions

A novel UV–Vis spectrophotometric method based on CPE preconcentration was developed for the determination of total vanadium. It can be categorized as an ionic-liquid-assisted, mixed-micelle-mediated, centrifuge-less CPE method, for which the abbreviation IL-MM-CL-CPE is proposed. Its analytical performance and practical applicability were demonstrated through the analysis of real samples and further evaluated using the RGBfast model.

The method exhibited high sensitivity, precision, repeatability, selectivity, simplicity, cost-effectiveness, and environmental friendliness. Owing to these favorable characteristics, the proposed procedure represents a competitive alternative to existing spectrophotometric methods for vanadium determination.

## Figures and Tables

**Table 1:** Optimized experimental parameters.

Parameter	Optimum
Wavelength *, nm	309
pH	3.3–3.4
Volume of the buffer, mL	1.0–1.5
Concentration of PG, mol L^−1^	1.43 × 10^−2^
Concentration of A336, mol L^−1^	7.1 × 10^−4^
Mass fraction of TX-114, %	2.1
Incubation time, min	30
Incubation temperature, °C	70
Cooling time (at −20 °C), min	25–30
Test tube capacity, mL	15.0
Volume of the sample, mL	14.0
Volume of the final (diluted) SRP, mL **	2.0

* Measurements providing maximum sensitivity. Alternative wavelengths are 342 nm and 436 nm. ** Diluted to the mark (2.0 mL) with 300 μL of C_2_H_5_OH and deionized water.

**Table 2:** Analytical characteristics.

Characteristic	Conventional Water Bath			Ultrasonic Bath		
	309 nm	364 nm	436 nm	309 nm	364 nm	436 nm
Linear range, ng mL^−1^	Up to 3.6	Up to 3.6	Up to 3.6	Up to 3.6	Up to 3.6	Up to 3.6
Intercept (*b*)	−0.008	−0.004	−0.001	−0.0011	−0.0004	−0.0004
Standard deviation (SD) of *b*	0.014	0.004	0.003	0.021	0.004	0.002
Slope (*a*)	0.630	0.141	0.081	0.626	0.141	0.081
SD of *a*	0.007	0.001	0.001	0.010	0.002	0.001
*R*-squared	0.9994	0.9997	0.9996	0.9987	0.9990	0.9993
SD of the blank (*n* = 10)	0.023	0.005	0.004	0.037	0.009	0.006
LOD *, ng mL^−1^	0.11	0.12	0.15	0.16	0.19	0.23
LOQ **, ng mL^−1^	0.36	0.39	0.52	0.53	0.64	0.76
Molar absorptivity coefficient (*ε*), L mol^−1^ cm^−1^	32 × 10^6^	7.2 × 10^6^	4.1 × 10^6^	32 × 10^6^	7.2 × 10^6^	4.1 × 10^6^
Sandell’s sensitivity, ng cm^−2^	1.6 × 10^−3^	7.1 × 10^−3^	1.24 × 10^−2^	1.6 × 10^−3^	7.1 × 10^−3^	1.24 × 10^−2^

* 3 × SD_blank_/a. ** 10 × SD_blank_/a.

**Table 3:** Effect of foreign ions on the determination of vanadium.

Ion	Added Compound	Ion-to-V Mass Ratio	Recovery, %		
			436 nm	362 nm	309 nm
Al(III)	Al_2_(SO_4_)_3_ · 18H_2_O	100	102	105	103
Ba(II)	Ba(NO_3_)_2_	2000 ^a^	101	103	101
Br^−^	NaBr	2000 ^a^	105	104	103
Ca(II)	Ca(NO_3_)_2_	1000 ^a^	104	105	105
Cd(II)	Cd(NO_3_)_2_ · 4H_2_O	2000 ^a^	97.0	100	99.3
Cl^−^	NaCl	2000 ^a^	101	102	101
Co(II)	Co(NO_3_)_2_	2000 ^a^	97.0	98.4	100
Cr(III)	CrK(SO_4_)_2_ · 12H_2_O	10	97.4	101	99.8
		50	102	106	104
Cr(VI)	K_2_CrO_4_	20	101	101	98.7
Cu(II)	Cu(NO_3_)_2_	1	109	112	113
F^−^	NaF	1000	97.2	98.7	96.1
Fe(III)	Fe_2_(SO_4_)_3_	5	102	105	104
		10 ^b^	97.7	99.9	101
		10 ^c^	105	105	102
Ge(IV)	GeO_2_	100	97.9	170	104
Hg(I)	Hg_2_(NO_3_)_2_	1000	99.2	106	106
Hg(II)	Hg(NO_3_)_2_	2000 ^a^	101	99.8	101
H_2_PO_4_^−^	NaH_2_PO_4_ · H_2_O	2000 ^a^	101	99.0	99.5
I^−^	KI	100	99.5	99.3	98.6
		200	103	109	108
Li(I)	Li_2_SO_4_	2000 ^a^	101	102	100
Mg(II)	MgSO_4_ · 7H_2_O	1000 ^a^	100	100	100
Mn(II)	MnSO_4_ · 5H_2_O	2000 ^a^	101	99.3	95.4
Mo(VI)	Na_2_MoO_4_	1	103	110	102
		5	112	115	98.2
		200	169	192	98.7
Ni(II)	NiSO_4_ · 7H_2_O	2000 ^a^	101	105	104
Pb(II)	Pb(NO_3_)_2_	2000 ^a^	103	101	102
Re(VII)	NH_4_ReO_4_	500	104	104	104
SCN^−^	KSCN	1000	97.4	102	102
Sr(II)	Sr(NO_3_)_2_	1000	101	103	101
		2000 ^a^	104	107	105
Tartrate^2−^	KNaC_4_H_4_O_6_·4H_2_O	1000	105	107	105
U(VI)	UO_2_(CH_3_CO_2_)_2_ · H_2_O	2000 ^a^	103	109	105
		1000	98	100	99
W(VI)	Na_2_WO_4_ · 2H_2_O	100	104	110	106
Zn(II)	ZnSO_4_ · 7H_2_O	1000	98.9	99.6	101

^a^ Higher mass ratios were not studied. ^b^ Masked with H_2_PO_4_^−^ (60 μg). ^c^ Masked with F^−^ (30 μg).

**Table 4:** Determination ^a^ of vanadium in dietary supplements.

Dietary Supplement	Manufacturer’s Declared Value per Capsule	Vanadium Found, μg		
		436 nm	362 nm	309 nm
Sample 1 ^b^	25 μg	24.48 ± 0.58	25.26 ± 0.60	24.51 ± 0.67
Sample 2 ^c^	2 mg	2024 ± 51	2016 ± 49	1995 ± 61

^a^ ±SD, *n* = 4. ^b^ Natural Factors^®^, Canada, Chromium & Vanadium. ^c^ Nutricost^®^, Vanadium (Vanadyl Sulfate with Chromium).

**Table 5:** Determination ^a^ of vanadium in water samples.

Water Samples	V Concentration, μg L^−1^		RSD, %	*R*, %
	Added	Found ^e^		
Sample 1 ^b^	0	0.65 ± 0.09	14.4	–
	0.91	1.63 ± 0.08	4.7	107
	1.82	2.44 ± 0.22	9.1	98.3
Sample 2 ^c^	0	0.75 ± 0.06	8.6	–
	0.91	1.58 ± 0.20	12.9	91.6
	1.82	2.61 ± 0.09	3.4	102.1
Sample 3 ^d^	0	5.72 ± 0.22	3.8	–
	0.91	6.62 ± 0.02	0.3	98.7
	1.82	7.55 ± 0.15	2.0	100.3

^a^ 309 nm. ^b^ Commercial table water. ^c^ Spring water. ^d^ Low-mineral-content mineral water, which is not recommended for daily consumption. ^e^ ±SD, *n* = 3.

**Table 6:** Determination ^a^ of vanadium in catalysts.

Catalyst Sample ^b^	Vanadium Found ^c^, %		RSD, %	
	Present Method	Alternative Method	Present Method	Alternative Method
Sample 1	2.82 ± 0.06	2.87 ± 0.11	2.1	3.9
Sample 2	2.35 ± 0.04	2.32 ± 0.10	1.7	4.3

^a^ 436 nm. ^b^ Spent catalyst material obtained from Holding KCM 2000, Plovdiv, Bulgaria. ^c^ ±SD, *n* = 4.

## Data Availability

Data are contained within the article.
